# Mental Health Burden of the Russian–Ukrainian War 2022 (RUW-22): Anxiety and Depression Levels among Young Adults in Central Europe

**DOI:** 10.3390/ijerph19148418

**Published:** 2022-07-09

**Authors:** Abanoub Riad, Anton Drobov, Martin Krobot, Natália Antalová, Muhammad Abdullatif Alkasaby, Aleš Peřina, Michal Koščík

**Affiliations:** 1 Department of Public Health, Faculty of Medicine, Masaryk University, 62500 Brno, Czech Republic; anton.drobov@med.muni.cz (A.D.); krobot@med.muni.cz (M.K.); natalia.antalova@med.muni.cz (N.A.); aperina@med.muni.cz (A.P.); 2 Department of Health Sciences, Faculty of Medicine, Masaryk University, 62500 Brno, Czech Republic; 3 Centre for Global Mental Health, London School of Hygiene & Tropical Medicine, Keppel St., London WC1E 7HT, UK; muhammad.alkasaby@lshtm.ac.uk

**Keywords:** anxiety, armed conflicts, Czech Republic, depression, patient health questionnaire, students, Ukraine

## Abstract

Armed conflicts are public health emergencies that affect human lives at multiple levels. The mental health of adolescents and young adults is at high risk during crisis settings; therefore, this cross-sectional survey-based study aimed to evaluate anxiety and depressive symptoms among university students in the Czech Republic following the Russian-Ukrainian war of 2022 (RUW-22). The study used standardized screening instruments; the Generalized Anxiety Disorder (GAD-7) for anxiety and the Patient Health Questionnaire (PHQ-9) for depression. Of 591 students who participated in this study, 67.7% were females, 68.2% held Czech citizenship, and 63.8% were enrolled in medical or healthcare programs. The participants were highly concerned about the RUW-22 news, with a mean score of 7.17 ± 2.50 (0–10). While 34% and 40.7% of the participants in this study manifested moderate to severe levels of anxiety and depression, respectively, the mental health burden of the RUW-22 was confirmed by the positive correlation between “feeling concerned”, GAD-7 (ρ = 0.454), and PHQ-9 (ρ = 0.326). Female gender, higher frequency of news following, and social media use were associated with higher levels of anxiety and depressive symptoms; thus, proposing them to be risk factors for psychological disorders following the RUW-22.

## 1. Introduction

The Russian-Ukrainian War (RUW-22) that began on 24 February 2022, has become one of the largest and fastest-growing humanitarian emergencies since World War II, with over 6.7 million Ukrainian refugees having fled their country to date [1]. Armed conflicts are broadly perceived as public health emergencies that require substantial levels of international cooperation, preparedness, and resilience to effectively respond to the escalating health needs of the affected communities [2,3,4]. The public’s mental health and well-being are affected by various disasters every year. Therefore, the World Health Organization (WHO) defines a disaster as “a sudden ecological phenomenon of sufficient magnitude to require external assistance” [5]. Man-made disasters include military conflicts, such as RUW-22 [6].

The individuals affected by war are at high risk of mental health complications, including posttraumatic disorder (PTSD), anxiety, and depression [7]. The various effects of war, such as damage to properties or other valuable assets, death of a close one, displacement of the family, lack of mental preparedness for disaster, lack of social support, and negative coping skills, are likely to adversely affect the mental health of Ukrainian people, including civilians and soldiers [8]. Peek (2008) [9] indicated that there are different behavioral, psychological, and emotional problems and instabilities found in children and women after any type of disaster. Emotional instability, stress reactions, anxiety, trauma, and other psychological symptoms are observed commonly after the disaster and other traumatic experiences. These psychological effects have a massive impact on the concerned individuals and communities [10]. In a systematic review of the war refugees’ mental health, Bogic et al., 2015 [11] found that psychological disorders, such as PTSD, depression, and unspecified anxiety disorders, tended to be highly prevalent among war refugees even after many years of resettlement.

Adolescents and young adults are depicted by the WHO as high-risk groups for mental disorders, including depression, anxiety, and behavioral disorders [12]. Therefore, the WHO’s comprehensive mental health action plan 2013–2030 insisted on the explicit inclusion of youth mental health within general and priority health policies [13]. Nevertheless, Ukrainians are supposed to be the most affected by the RUW-22 as first-hand responders to the ongoing crisis; the psychological impact of the war on neighboring populations, especially in central Europe, cannot be omitted. In an earlier incident, namely, the Chernobyl nuclear disaster of 1986, long-term physical and mental disorders were reported by local communities in neighboring countries, e.g., Belarus and the Czech Republic [14,15].

The North Atlantic Treaty Organization (NATO) member states, including the Czech Republic, responded immediately and firmly to the RUW-22 by imposing a package of sanctions against the Russian administration [16]. Moshagen et al., 2022, attempted to explore psychological reactions of citizens in some NATO states, i.e., Germany, Poland, the United States (US), and the United Kingdom (UK), because upholding these unified political actions is questionable and it depends on citizens’ perceptions and opinions [17]. While high levels of anxiety, anger, perceived threat, and empathy towards Ukrainian people were reported, a strong yet variable desire to escalate sanctions against Russia was found [17]. Notably, Polish citizens had the strongest desire to impose severe sanctions against Russia, which can be attributed to several factors, such as geographical proximity of Poland to the conflict zone and receiving the largest share of Ukrainian refugees [17].

In spite of the fact that the Czech Republic does not share borders with Ukraine, it hosted about half a million Ukrainian refugees thus far [18]. Additionally, the Russian invasion of Ukrainian territories may remind the Czech citizens of the assault that happened in 1968 when thousands of Soviet tanks entered the country to terminate the liberalization movement known as the Prague Spring [19].

The overarching goal of this study was to explore the mental health burden of the RUW-22 in the Czech Republic among young adults. The primary objective was to assess anxiety and depression among Czech university students; the secondary objectives were (a) to evaluate the correlation between the RUW-22 news and anxiety and depression levels and (b) to explore the risk factors for anxiety and depression among the target population.

## 2. Materials and Methods

### 2.1. Design

A cross-sectional survey-based study was designed in March 2022 in response to the emerging military conflict in Ukraine (RUW-22) in order to evaluate its mental health burden. The study was conducted and reported according to the Strengthening the Reporting of Observational Studies in Epidemiology (STROBE) guidelines for cross-sectional studies [20].

### 2.2. Setting

The present study utilized a bilingual self-administered questionnaire (SAQ) offered in Czech and English. The SAQ was designed and administered digitally using KoBoToolbox (Harvard Humanitarian Initiative, Cambridge, MA, USA, 2022) [21]. The potential participants were invited to fill in the digital SAQ using shortened Uniform Resource Locators (URLs) and quick response (QR) codes publicized through official social media channels of Masaryk University, e.g., Twitter, Instagram, and Facebook. Additionally, promotional printed posters were placed in the Faculty of Medicine (Masaryk University) to increase students’ awareness of the survey and participation rate. Student unions and organizations were approached to promote the survey among their members. Individual academics were contacted to promote the SAQ among their students in other universities, e.g., Charles University, University of Defence, and Mendel University in Brno.

### 2.3. Population

The target population of this study were Czech university students who were recruited to participate in this study through a nonrandom sampling strategy (snow-balling technique). Inclusion criteria were (i) being a full-time/part-time student enrolled in any study program at any Czech university/higher education institute, and (ii) being able to communicate fluently either in Czech or English. Exclusion criteria were (i) Erasmus or exchange students, and (ii) the students enrolled in preparatory courses. The participants were not offered any incentives to take part, and their willingness was not coerced by any form of threats.

The minimum sample size was calculated using Epi-Info version 7.2.5 (CDC. Atlanta, GA, USA, 2021) following the assumptions of confidence level (CI) 95%, an acceptable margin of error of 5%, expected frequency of 50%, target population (N) of 300,000, and postulated response rate generated by careless or insufficient effort (C/IE) of 10% [22]. The required sample size was 423 participants.

### 2.4. Instrument

The SAQ used in this study consisted of closed-ended questions about participants’ demographic characteristics, i.e., gender, age, nationality, pre-university residence, current region, study field, and university. In the second section, there was an 11-point hedonic scale to assess participants’ level of being concerned by the RUW-22—“How much are you concerned by the news of the Russian-Ukrainian War?”, where 0 represented “not concerned at all” and 10 represented “extremely concerned”. The frequency of following war news and the used news outlets were assessed by multiple-choice questions.

The content validity of the proposed SAQ was evaluated by a panel of experts in public health, clinical psychology, and psychiatry. The panel recommended a few modifications for the wording of certain items, which were modified before the SAQ proceeded to the reliability testing stage. The test-retest reliability was evaluated through a group of volunteer students (*n* = 10) who filled in the SAQ twice with at least two weeks of patency period. The proposed SAQ had substantial reliability denoted by a mean Cohen’s kappa coefficient of 0.721 ± 0.214 (Table 1).

The third section of the SAQ aimed to evaluate self-reported symptoms of anxiety using the instrument of Spitzer et al., 2006 (General Anxiety Disorder: GAD-7), while the fourth section aimed to evaluate the severity of depressive symptoms using the instrument of Kroenke et al., 2001 (Patient Health Questionnaire: PHQ-9) [24,25]. According to the GAD-7 developers, a score of 0–4 indicates minimal anxiety, 5–9—mild anxiety, 10–14—moderate anxiety, and ≥15—severe anxiety [24,26]. According to the PHQ-9 developers, a score of 0–4 indicates no or minimal depression, 5–9—mild depression, 10–14—moderate depression, 15–19—moderately severe depression, and 20–27—severe depression [25,27]. Both instruments (GAD-7 and PHQ-9) were translated from English to Czech by Pfizer (Pfizer Inc. New York City, NY, USA), and the translated versions were used in this study [28]. The translated versions of GAD-7 and PHQ-9 produced by Pfizer are developed by the MAPI Research Institute (Lyon, France) that utilizes a standard translation methodology comprising of conceptual analysis, forward and backward translation, cognitive debriefing, proofreading, and finalization [29].

### 2.5. Ethics

The study was exempted from the ethical review process according to the opinion of the Ethics Committee, Faculty of Medicine, Masaryk University, as it was an entirely observational study with no anticipated harms. Informed consent was submitted electronically by all participants prior to their participation in this study as a prerequisite for displaying the SAQ items. Identifying personal data were not collected from the participants to keep their identities anonymous. The participants were able to leave the study at any time without the need to justify their decision, and no responses were recorded until the participant finalized the entire SAQ and confirmed to send out their responses.

### 2.6. Analyses

Initially, normal distribution of numerical variables, such as age, level of “feeling concerned”, GAD-7 score, and PHQ-9 score, was evaluated using the Shapiro–Wilk test that indicated that non-parametric analysis should be used subsequently. Descriptive statistics were performed to summarize qualitative variables using frequencies (*n*) and percentages (%) and numerical variables using means and standard deviations (*µ* ± *SD*). Inferential statistics were performed to explore the association between demographic variables and level of “feeling concerned”, news following frequency and outlets, and psychological status. Mann–Whitney test (*U*), Kruskal–Wallis test (*H*), and Spearman correlation were used with a confidence interval (*CI*) of 95% and a significance level (*Sig*.) of ≤0.05. The statistical analyses were performed using Statistical Package for the Social Sciences (SPSS) version 28.0 (SPSS Inc. Chicago, IL, USA, 2020) and the R-based open software Jamovi [30,31].

## 3. Results

### 3.1. Sample Characteristics

A total of 591 students participated in this study, of which 400 (67.7%) were females and 335 (56.7%) were aged 22 years or below. The most contributing region was South Moravian Region (59.9%), followed by Moravian Silesian Region (7.8%), Vysočina Region (6.8%), and Zlín Region (5.4%). Regarding their pre-university residence, 38.6% of the participants came from cities with >100,000 inhabitants, while 30.6% came from towns/villages with ≤10,000 inhabitants and the rest came either from cities with >10,000 or >500,000 inhabitants.

The majority of participants had Czech nationality (68.2%), followed by Slovak (22%) and other nationalities (9.8%). Masaryk University was the most represented higher education institution in this study (83.6%), followed by Charles University (4.6%), University of Defence (4.1%), and Mendel University in Brno (1.7%). Regarding study fields, medical and healthcare sciences were the most common fields (63.8%), followed by education and social care (9.3%), social sciences (9.1%), and natural sciences (5.9%) (Supplementary File Table S1).

### 3.2. Feeling Concerned by the RUW-22

In response to the question about feeling concerned by the RUW-22, the participants’ mean score was 7.17 ± 2.50 and ranged between 0 and 10. Female participants had a significantly (*Sig*. < 0.001) higher mean score than males, 7.51 vs. 6.45, respectively. Likewise, participants older than 22 years had a significantly (*Sig*. < 0.001) higher level of “feeling concerned”. There was no statistically significant difference between participants of different pre-university residences in terms of “feeling concerned” score. Slovak students had a significantly (*Sig*. < 0.001) higher score than Czech students, and international students had the lowest score. Across study fields, the highest score was achieved by the students of agriculture, forestry, and veterinary sciences (9.00 ± 1.41), followed by arts and humanities (8.78 ± 1.20) and natural sciences (7.80 ± 2.01), while the students of military sciences had the lowest score (4.68 ± 2.58) (Table 2).

### 3.3. Following of RUW-22 News

When asked about the frequency of following RUW-22 news, 11.8% reported checking the news every couple of hours, 6.6% three times a day, 15.6% twice a day, and 27.7% at least once a day. The most commonly used news outlets were digital news portals (82.8%), followed by social media networks (72.4%) and television (37.5%) (Table 3).

### 3.4. Generalized Anxiety Disorder (GAD-7) and Depression (PHQ-9)

In GAD-7, feeling nervous (Q1) had the largest proportion of “several days” answers—42.3%, followed by becoming easily annoyed (Q6)—40.8%, and feeling afraid of something awful (Q7)—38.9%. In addition, worrying too much about different things (Q3) had the largest proportion of “more than half of the days” answer—24.4%, followed by having trouble relaxing (Q4)—24% and feeling nervous (Q1)—21%. Regarding the “nearly every day” answer, it was the most common in feeling nervous (Q1) and least in being so restless (Q5) (Supplementary File Table S2).

In PHQ-9, having little interest or pleasure in doing things (Q1) had the largest proportion of “several days” answer—40.4%, followed by feeling tired with low energy (Q4)—35%, and feeling down or depressed (Q2)—34.2%. In addition, feeling tired with low energy (Q4) had the largest proportion of “more than half of the days” answer—28.1%, followed by having little interest or pleasure in doing things (Q1)—17.9% and having poor appetite or overeating (Q5)—17.3%. The “nearly every day” answer was the most common in feeling tired (Q4) and the least common in moving or speaking so slowly (Q8). The ninth question about suicidal ideation was answered by 21 students (3.6%) as “nearly every day” (Supplementary File Table S3).

### 3.5. GAD-7 and PHQ-9 Scores

The mean GAD-7 score was 7.86 ± 5.32, with 22.3% and 13.7% of the participants exhibiting moderate and severe anxiety symptoms, respectively. Females had a significantly higher GAD-7 score than their male counterparts (8.64 vs. 6.11; *Sig*. < 0.001). No statistically significant differences were found between participants of different age groups (*Sig*. = 0.798) or nationalities (*Sig*. = 0.113). The students who came from cities with >500,000 inhabitants had the highest GAD-7 scores (8.30 ± 6.09) compared with other pre-university residences.

The students of education and social care (9.51 ± 5.39), social sciences (9.69 ± 5.66), and arts and humanities (12.67 ± 4.98) had significantly higher GAD-7 scores than the average score of the whole sample. On the other hand, students of military sciences had the lowest GAD-7 scores (2.96 ± 3.50). The students who had >7 scores of “feeling concerned” had a significantly higher GAD-7 scores than those who had ≤7 scores (9.65 vs. 5.56; *Sig*. < 0.001). Following the news every couple of hours (11.03 ± 5.84) and using social media networks (8.38 ± 5.32) were associated with the highest GAD-7 scores (Table 4).

The mean PHQ-9 score was 8.66 ± 6.29, with 22%, 11%, and 7.1% of the participants exhibiting moderate, moderately severe, and severe depressive symptoms, respectively. Females had a significantly higher PHQ-9 score than their male counterparts (9.38 vs. 7.03; *Sig*. < 0.001). No statistically significant differences were found between participants of different age groups (*Sig*. = 0.503) or nationalities (*Sig*. = 0.536). The students who came from cities with >500,000 inhabitants had the highest PHQ-9 scores (8.92 ± 6.79) compared with other pre-university residences.

**Figure 1:**
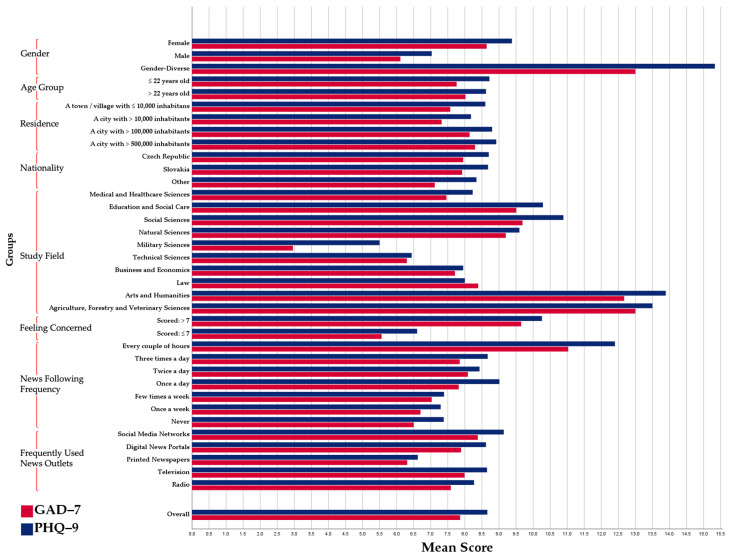
Mean GAD-7 and PHQ-9 scores among Czech university students participating in the RUW-22 survey stratified by groups, April–May 2022, (*n* = 591).

The students of education and social care (10.29 ± 6.59), social sciences (10.89 ± 6.56), and arts and humanities (13.89 ± 7.61) had significantly higher PHQ-9 scores than the average score of the whole sample. On the other hand, the students of military sciences (5.50 ± 5.62) and technical sciences (6.44 ± 5.91) had the lowest PHQ-9 scores. The students who had >7 scores of “feeling concerned” had a significantly higher PHQ-9 scores than those who had ≤7 scores (10.26 vs. 6.60; *Sig*. < 0.001). Following the news every couple of hours (12.40 ± 7.08) and using social media networks (9.14 ± 6.29) were associated with the highest PHQ-9 scores (Figure 1).

### 3.6. Correlation Analysis

Non-parametric correlation revealed that “feeling concerned” was moderately correlated with frequency of news following (ρ = 0.445; *Sig*. < 0.001) and GAD-7 score (ρ = 0.454; *Sig*. < 0.001) and weakly correlated with PHQ-9 score (ρ = 0.326; *Sig*. < 0.001). GAD-7 and PHQ-9 scores were strongly correlated (ρ = 0.764; *Sig*. < 0.001) (Table 5).

## 4. Discussion

The present study revealed that Czech university students were highly concerned about the Russian–Ukrainian War 2022 (RUW-22) news, with a mean score of 7.17 ± 2.50 (0–10). Most participants (61.7%) reported following the war news at least once a day, with digital news portals being the most utilized outlet (82.8%), followed by social media networks (72.4%) and television (37.5%). More than one-third of the participants exhibited moderate (22.3%) and severe (13.7%) anxiety, and more than two-fifths exhibited moderate (22%), moderately severe (11%), and severe (7.1%) depression according to GAD-7 and PHQ-9 scales.

The “feeling concerned by the RUW-22” level was significantly higher among female participants than their male counterparts. The environment can modify the gender-based differences in risk perception, as females tend to exhibit higher levels of concern than males when examined in non-stressed environments. However, in stressed environments, females and males exhibit comparable levels of concern [32,33]. Females’ predisposition to “feeling concerned” can be explained by the fact that the Czech Republic is not a conflict area per se. In the US, females were found to be more fearful of possible terror attacks; hence they had higher levels of information-seeking behaviors [34].

Additionally, the level of “feeling concerned by the RUW-22” was significantly higher among our older students (>22 years old), which is consistent with previous studies. Numerous studies found perceived risk to be positively correlated with the age of the participants [35,36]. Kim et al., 2018, demonstrated that older adolescents had significantly higher risk perception levels than younger adolescents [37].

As compared with the students from the Czech Republic, the students from Slovakia had a higher level of “feeling concerned” (7.05 vs. 7.68; *Sig*. < 0.001, respectively). This finding can be explained by the fact that Slovakia is a bordering country of Ukraine which made it a haven for refugee influx, with 446,755 Ukrainian refugees received up until May 24th, representing 8.2% of the country’s population; at the same time, the Czech Republic granted 354,631 emergency visas to Ukrainian refugees up until May 25th, representing only 3.4% of the country’s population [38,39]. Further multi-country studies are required to verify whether geographical proximity has a role in modifying the levels of perceived risk.

In our study, 61.7% of the participants reported following the RUW-22 news at least once daily, thus suggesting that the frequency of following war news might be a resultant of “feeling concerned” and a predictor for psychologic disorders, i.e., anxiety and depression. The correlation test revealed that the frequency of news following was moderately correlated with “feeling concerned” (ρ = 0.445) and weakly correlated with GAD-7 (ρ = 0.198) and PHQ-9 (ρ = 0.181) scores. Likewise, Malka et al., 2015, found that news consumption frequency, which increased during the time of military conflict, was also positively correlated with the level of worry (r = 0.18) [40,41]. Among Israeli adults, the frequency of news consumption increased significantly during the 2014 Gaza war compared with the pre-war period, and it was associated with anxiety, hyperarousal, and sleeping disorders [42]. Recently, Danielle et al., 2022, revealed that frequency of exposure to COVID-19 pandemic-related news was a predictor for greater anxiety and depression among US adults during the first wave in 2020 [43].

Digital news portals (82.8%) and social media networks (72.4%) were the most commonly used news outlets by our participants. Multiple prior studies found that social media platforms had been the most frequently used information source by Generation Z, even for health-related information and recommendations [44,45]. The current finding that digital news portals outperformed social media can be attributed to the questionable credibility of social media networks that may facilitate the dispersion of fake news, especially during conflict times [46,47,48]. The participants who reported using social media networks had significantly higher levels of anxiety (8.38 ± 5.32) and depression (9.14 ± 6.29) compared to the students who used other news outlets. One explanation for this finding could be attributed to the type of content conveyed by social media platforms that could be more emotional than official news portals and television. Frequent exposure to social media content was associated with higher odds of anxiety among Chinese adults during the COVID-19 outbreak in early 2020 [49,50]. Furthermore, according to the study conducted by the Ministry of the Interior of the Czech Republic at the end of 2019, the most traced source of information by the youngest age groups in the Czech Republic was social media [51]. Television and radio were the most confidential source of information, while in our study they were consumed by the minority of participants, i.e., TV (37%) and radio (9%). The confidentiality of social media has not been analyzed in that report but disinformation narratives about Russian activities in the Crimea peninsula and in most regions of Ukraine were already included [51].

Overall, the mean GAD-7 score of our participants was 7.86 ± 5.32, which was significantly (*Sig*. < 0.001) higher among females (8.64 ± 5.29) than males (6.11 ± 4.96). Females are more susceptible to anxiety disorders in emergency settings, including generalized anxiety disorder. Among the internally displaced families in Ukraine between 2014 and 2019, mothers had higher GAD-7 scores than fathers [52]. Elhadi et al., 2020, found that in response to the civil war in Libya, female medical students had higher scores of GAD-7 than their male colleagues [53]. Likewise, during the Syrian civil war, female adults were 1.5 times more likely to suffer from anxiety disorders than males assessed by GAD-7 [54]. Additionally, female Libyan adults had a significantly higher GAD-7 score than their male counterparts following the COVID-19 lockdown [55]. However, Slovak students had higher levels of “feeling concerned”; there was no significant difference between our participants of different nationalities in terms of GAD-7 or PHQ-9 scores. No statistically significant difference in GAD-7 scores was found across age groups or pre-university residences, thus confirming the null hypothesis that age and pre-university residence would have no impact on students’ reactions to the RUW-22 news.

The mean PHQ-9 scores of our participants were 8.66 ± 6.29, which was significantly (*Sig*. < 0.001) higher among females (9.38 ± 6.41) than males (7.03 ± 5.68). Similar to anxiety disorders, females are also more susceptible to depressive symptoms during public health emergencies [56]. Fanaj et al., 2021, found that female adults in Kosovo had higher PHQ-9 scores than their male counterparts during the COVID-19 outbreak [57]. Depressive symptoms, as summarized by the PHQ-9 scores, were not significantly different across age groups, nationalities, or pre-university residences.

When asked about suicidal ideations, 3.6% of the students responded that they have suicidal thoughts nearly every day and 4.8% for more than half of the days. This is a warning sign that requires more attention given that suicide is among the leading causes of death in this age group and that experiencing disasters and conflicts is strongly associated with suicidal behaviors [58,59].

The positive correlation between “feeling concerned” and GAD-7 (ρ = 0.454) and PHQ-9 (ρ = 0.326) scores indicated that the current war could have a role in worsening/exacerbating anxiety and depressive symptoms among our participants. In Spain, being less concerned with COVID-19 pandemic updates was positively correlated with lower GAD-7 and PHQ-9 scores, thus indicating the role of pandemic news in exacerbating community anxiety and depression levels [60].

Regarding the representativeness of our sample, 67.7% of the participants were females, reflecting the female predominance of higher education in the Czech Republic. In addition, 31.8% of our participants were foreigners, with Slovaks being the most common foreign nationality (22%), followed by Germans, Russians, and Ukrainians. In its 2021 report, the Czech Statistical Office (CZSO) revealed that 55.5% of all university students in the Czech Republic were females and 16.7% were foreigners, with Slovaks being the most common foreign nationality followed by Russians and Ukrainians [61].

### 4.1. Strengths

To the best of our knowledge, this study is the first to evaluate the potential mental health burden of the RUW-22 in the short-term interval. It also utilized standardized instruments (GAD-7 and PHQ-9) that were widely used in the last two decades, manifesting high levels of screening accuracy, especially in crisis settings. The recruited sample, however, was selected non-randomly, reflecting the target population’s demographic characteristics, i.e., Czech university students. The participants’ identities were anonymous to limit information bias and Hawthorne’s effect.

### 4.2. Limitations

The present study has several limitations that are related to its design. As a cross-sectional study, selection bias, especially self-selection, is unavoidable; therefore, our findings regarding the prevalence of anxiety and depression levels should be interpreted cautiously as over-estimation cannot be omitted in our study setting. The instruments used in this study (GAD-7 and PHQ-9) are not meant to provide a clinical diagnosis, although they perform better for screening purposes. Therefore, the results of this study should be used as indicative of the mental health status of Czech university students. The level of “feeling concerned” is a dynamic concept which can rapidly change over time; therefore, we found a low kappa coefficient during the test-retest phase among few subjects. Another limitation for the construct of “feeling concerned” is that it was measured by an 11-point scale which might be challenging for some participants.

### 4.3. Implications

In light of the current findings, it is imperative for public health authorities in central European countries, including the Czech Republic, to consider the mental health burden of the RUW-22 on their young adult populations. Female gender, higher frequency of news following, and use of social media were associated with higher levels of anxiety and depression, thus suggesting them to be the high-risk groups for community-level interventions targeting young adults. Future studies should evaluate the reasons for “feeling concerned” about the ongoing war and compare the mental health status of youth in various European countries.

## 5. Conclusions

Within the limitations of this study, Czech university students were found to be highly concerned about the Russian–Ukrainian War 2022 (RUW-22) news. More than one-third of the participants manifested moderate to severe levels of anxiety and depression; and the psychological burden of the RUW-22 was indicated by the moderate positive correlation between “feeling concerned”, GAD-7, and PHQ-9. Female gender, higher frequency of news following, and social media use were associated with higher levels of anxiety and depressive symptoms; thus, suggesting them to be risk factors for psychological disorders following the RUW-22.

## Figures and Tables

**Table 1:** The results of test-retest reliability of the second section items.

ID	*κ*	ID	*κ*
1	0.700	6	0.705
2	0.667	7	0.836
3	0.836	8	1.000
4	0.700	9	0.385
5	1.000	10	0.385

Cohen’s kappa statistic (*κ*): 0.01–0.20 as none–light; 0.21–0.40 as fair; 0.41–0.60 as moderate; 0.61–0.80 as substantial; and 0.81–1.00 as perfect agreement [23].

**Table 2:** Level of “Feeling Concerned by the RUW-22” among Czech university students participating in the RUW-22 survey, April–May 2022, (*n* = 591).

Variable	Outcome	Feeling Concerned 7.17 ± 2.50 (0–10)	*Sig*.
Gender	Female	7.51 ± 2.24	<0.001
	Male	6.45 ± 2.88	
	Diverse	7.67 ± 1.53	0.936
Age Group	≤22 years old	7.00 ± 2.50	0.016
	>22 years old	7.42 ± 2.49	
Residence	A town/village with ≤10,000 inhabitants	7.12 ± 2.41	0.356
	A city with >10,000 inhabitants	6.80 ± 2.75	
	A city with >100,000 inhabitants	7.45 ± 2.28	
	A city with >500,000 inhabitants	6.97 ± 2.89	
Nationality	Czech Republic	7.05 ± 2.56	0.029
	Slovakia	7.68 ± 2.04	
	Other	6.91 ± 2.91	0.743
Study Field	Medical and Healthcare Sciences	7.23 ± 2.41	0.900
	Education and Social Care	7.09 ± 2.40	0.613
	Social Sciences	7.52 ± 2.78	0.079
	Natural Sciences	7.80 ± 2.01	0.164
	Military Sciences	4.68 ± 2.58	<0.001
	Technical Sciences	6.26 ± 3.01	0.141
	Business and Economics	7.38 ± 2.46	0.677
	Law	6.83 ± 2.81	0.762
	Arts and Humanities	8.78 ± 1.20	0.041
	Agriculture, Forestry and Veterinary Sciences	9.00 ± 1.41	0.254

Mann–Whitney test (*U*) and Kruskal–Wallis (*H*) were used with a significance level (*Sig*.) ≤ 0.05.

**Table 3:** News following frequency and outlets among Czech university students participating in the RUW-22 Survey, April–May 2022, (*n* = 591).

Variable	Outcome	Frequency (*n*)	Percentage (*%*)
How frequently do you check (or are exposed to) the news of the Russian–Ukrainian War?	Every couple of hours	70	11.8
	Three times a day	39	6.6
	Twice a day	92	15.6
	Once a day	164	27.7
	Few times a week	106	17.9
	Once a week	86	14.6
	Never	34	5.8
What resources do you use to follow the news of the Russian–Ukrainian War?	Social Media Networks	403	72.4
	Digital News Portals	461	82.8
	Printed Newspapers	13	2.3
	Television	209	37.5
	Radio	51	9.2

**Table 4:** GAD-7 and PHQ-9 scores among Czech university students participating in the RUW-22 survey, April–May 2022, (*n* = 591).

Variable	Outcome	Frequency (*n*)		Percentage (*%*)	
GAD-7 Score Level	Minimal: 0–4	187		31.6	
	Mild: 5–9	191		32.3	
	Moderate: 10–14	132		22.3	
	Severe: >14	81		13.7	
PHQ-9 Score Level	None–Minimal: 0–4	186		31.5	
	Mild: 5–9	168		28.4	
	Moderate: 10–14	130		22.0	
	Moderately Severe: 15–19	65		11.0	
	Severe: 20–27	42		7.1	
**Variable**	**Outcome**	**GAD-7** 7.86 ± 5.32 (0–21)	*Sig*.	**PHQ-9** 8.66 ± 6.29 (0–27)	*Sig*.
Gender	Female	8.64 ± 5.29	<0.001	9.38 ± 6.41	<0.001
	Male	6.11 ± 4.96		7.03 ± 5.68	
	Diverse	13.00 ± 1.00	0.067	15.33 ± 5.13	0.061
Age Group	≤22 years old	7.76 ± 5.12	0.798	8.72 ± 6.07	0.503
	>22 years old	8.02 ± 5.57		8.62 ± 6.58	
Residence	A town/village with ≤10,000 inhabitants	7.57 ± 5.21	0.483	8.60 ± 6.40	0.865
	A city with >10,000 inhabitants	7.32 ± 5.21		8.18 ± 5.87	
	A city with >100,000 inhabitants	8.14 ± 5.11		8.80 ± 6.19	
	A city with >500,000 inhabitants	8.30 ± 6.09		8.92 ± 6.79	
Nationality	Czech Republic	7.95 ± 5.28	0.987	8.70 ± 6.36	0.768
	Slovakia	7.92 ± 5.01		8.68 ± 5.89	
	Other	7.12 ± 6.25	0.113	8.34 ± 6.74	0.536
Study Field	Medical and Healthcare Sciences	7.46 ± 4.91	0.063	8.23 ± 5.97	0.064
	Education and Social Care	9.51 ± 5.39	0.019	10.29 ± 6.59	0.047
	Social Sciences	9.69 ± 5.66	0.009	10.89 ± 6.56	0.006
	Natural Sciences	9.20 ± 5.47	0.145	9.60 ± 6.90	0.422
	Military Sciences	2.96 ± 3.50	<0.001	5.50 ± 5.62	0.003
	Technical Sciences	6.30 ± 5.293	0.061	6.44 ± 5.91	0.043
	Business and Economics	7.71 ± 5.72	0.839	7.95 ± 6.29	0.608
	Law	8.39 ± 5.65	0.764	8.00 ± 5.48	0.749
	Arts and Humanities	12.67 ± 4.98	0.010	13.89 ± 7.61	0.032
	Agriculture, Forestry and Veterinary Sciences	13.00 ± 11.31	0.405	13.50 ± 13.44	0.533
Feeling Concerned	Scored: ≤7	5.56 ± 4.75	<0.001	6.60 ± 5.65	<0.001
	Scored: >7	9.65 ± 5.05		10.26 ± 6.30	
News Following Frequency	Every couple of hours	11.03 ± 5.84	<0.001	12.40 ± 7.08	0.001
	Three times a day	7.85 ± 4.74		8.67 ± 6.12	
	Twice a day	8.09 ± 5.13		8.43 ± 6.12	
	Once a day	7.82 ± 5.14		9.01 ± 6.18	
	Few times a week	7.03 ± 4.94		7.39 ± 5.92	
	Once a week	6.70 ± 5.18		7.29 ± 5.46	
	Never	6.50 ± 5.42		7.38 ± 6.05	
Frequently Used News Outlets	Social Media Networks	8.38 ± 5.32	0.001	9.14 ± 6.29	0.007
	Digital News Portals	7.89 ± 5.33	0.512	8.62 ± 6.21	0.397
	Printed Newspapers	6.31 ± 6.07	0.181	6.62 ± 6.38	0.136
	Television	7.99 ± 5.21	0.828	8.65 ± 6.17	0.858
	Radio	7.59 ± 4.77	0.698	8.27 ± 5.66	0.769

Mann–Whitney test (*U*) and Kruskal–Wallis test (*H*) were used with a significance level (*Sig*.) ≤ 0.05.

**Table 5:** Correlation between “Feeling Concerned”, news following frequency, GAD-7, and PHQ-9 among Czech university students participating in the RUW-22 survey, April–May 2022, (*n* = 591).

		Feeling Concerned	News Following Frequency	GAD-7	PHQ-9
Feeling Concerned	ρ	1.000			
	*Sig.*	N/A			
Following News Frequency	ρ	0.445	1.000		
	*Sig.*	<0.001	N/A		
GAD-7	ρ	0.454	0.198	1.000	
	*Sig.*	<0.001	<0.001	N/A	
PHQ-9	ρ	0.326	0.181	0.764	1.000
	*Sig.*	<0.001	<0.001	<0.001	N/A

Spearman’s correlation was used with a significance level (*Sig*.) ≤ 0.05. To interpret Spearman’s correlation coefficient values (ρ): 0–0.10 (negligible correlation), 0.10–0.39 (weak correlation), 0.40–0.69 (moderate correlation), 0.70–0.89 (strong correlation), and 0.90–1 (very strong correlation).

## Data Availability

The data that support the findings of this study are available from the corresponding author upon reasonable request.

## Supplementary Materials

The following supporting information can be downloaded at: https://www.mdpi.com/article/10.3390/ijerph19148418/s1, Table S1. Demographic characteristics of Czech university students participating in the RUW-22 survey, April–May 2022, (*n* = 591); Table S2. Generalized Anxiety Disorder-7 (GAD-7) Responses of Czech university students participating in the RUW-22 survey, April–May 2022, (*n* = 591); Table S3. Patient Health Questionnaire-9 (PHQ-9) responses of Czech university students participating in the RUW-22 survey, April–May 2022, (*n* = 591). Reference [62] is cited in the supplementary materials.
