# Supplementary material for: Mental Health Burden of the Russian–Ukrainian War 2022 (RUW-22): Anxiety and Depression Levels among Young Adults in Central Europe
Source: Int J Environ Res Public Health. 2022 Jul 9;19(14):8418. doi: 10.3390/ijerph19148418 (PMC9318466; doi:10.3390/ijerph19148418)
Supplement: Supplementary file 1 [file ijerph-19-08418-s001.zip › ijerph-1778027-supplementary.pdf]

International Journal of
Environmental Research and Public Health

Table S1. Demographic Characteristics of Czech Universities Students Participating in the RUW-22 Survey, April – May 2022, (*n* = 591).

Variable	Outcome	Frequency (n)	Percentage (%)
**Gender**	Female	400	67.7
	Male	188	31.8
	Diverse	3	0.5
**Age Group**	≤ 22 years old	335	56.7
	> 22 years old	255	43.1
	Missed	1	0.2
**Region**	South Moravian Region	354	59.9
	Moravian-Silesian Region	46	7.8
	Vysočina Region	40	6.8
	Zlín Region	32	5.4
	Central Bohemian Region	30	5.1
	Hradec Králové Region	23	3.9
	Olomouc Region	22	3.7
	South Bohemian Region	15	2.5
	Pardubice Region	15	2.5
	Ústí nad Labem Region	5	0.8
	Liberec Region	4	0.7
	Plzen Region	4	0.7
	Karlovy Vary Region	1	0.2
**Residence**	A town / village with ≤ 10,000 inhabitants	181	30.6
	A city with > 10,000 inhabitants	91	15.4
	A city with > 100,000 inhabitants	228	38.6
	A city with > 500,000 inhabitants	91	15.4
**Nationality**	Czech Republic	403	68.2
	Slovakia	130	22.0
	Germany	12	2.0
	Russia	9	1.5
	Ukraine	8	1.4
	Israel	4	0.7
	Italy	4	0.7
	Belarus	3	0.5
	India	2	0.3
	Portugal	2	0.3
	Spain	2	0.3
	Sweden	2	0.3
	United Kingdom	2	0.3
	Colombia	1	0.2
	Croatia	1	0.2
	Cyprus	1	0.2
	France	1	0.2
	Kazakhstan	1	0.2
	Norway	1	0.2
	South Africa	1	0.2
	Sri Lanka	1	0.2
**University**	Masaryk University	494	83.6
	Charles University	27	4.6

	University of Defence	24	4.1
	Mendel University in Brno	10	1.7
	Tomas Bata University in Zlín	8	1.4
	University of Chemistry and Technology	8	1.4
	University of Ostrava	5	0.8
	Other	15	2.5
**Study Field**	Medical and Healthcare Sciences	377	63.8
	Education and Social Care	55	9.3
	Social Sciences	54	9.1
	Natural Sciences	35	5.9
	Military Sciences	28	4.7
	Technical Sciences	27	4.6
	Business and Economics	21	3.6
	Law	18	3.0
	Arts and Humanities	9	1.5
	Agriculture, Forestry and Veterinary Sciences	2	0.3

Table S2. Generalized Anxiety Disorder – 7 (GAD-7) Responses of Czech Universities Students Participating in the RUW-22 Survey, April – May 2022, (*n* = 591)

Variable	Outcome	Frequency (*n*)	Percentage (%)
**Q1: Feeling nervous, anxious, or on edge**	Not at all	113	19.1
	Several days	250	42.3
	More than half of the days	124	21.0
	Nearly every day	104	17.6
**Q2: Not being able to stop or control worrying**	Not at all	214	36.2
	Several days	224	37.9
	More than half of the days	105	17.8
	Nearly every day	48	8.1
**Q3: Worrying too much about different things**	Not at all	123	20.8
	Several days	214	36.2
	More than half of the days	144	24.4
	Nearly every day	110	18.6
**Q4: Having trouble relaxed**	Not at all	139	23.5
	Several days	219	37.1
	More than half of the days	142	24.0
	Nearly every day	91	15.4
**Q5: Being so restless that it is hard to sit still**	Not at all	347	58.7
	Several days	146	24.7
	More than half of the days	72	12.2
	Nearly every day	26	4.4
**Q6: Becoming easily annoyed or irritable**	Not at all	182	30.8
	Several days	241	40.8
	More than half of the days	117	19.8
	Nearly every day	51	8.6
**Q7: Feeling afraid, as if something awful might happen**	Not at all	183	31.0
	Several days	230	38.9
	More than half of the days	110	18.6
	Nearly every day	68	11.5

Table S3. Patient Health Questionnaire – 9 (PHQ-9) Responses of Czech Universities Students Participating in the RUW-22 Survey, April – May 2022, (*n* = 591)

Variable	Outcome	Frequency (*n*)	Percentage (%)
**Q1: Little interest or pleasure in doing things**	Not at all	185	31.3
	Several days	239	40.4
	More than half of the days	106	17.9
	Nearly every day	61	10.3
**Q2: Feeling down, depressed, or hopeless**	Not at all	234	39.6
	Several days	202	34.2
	More than half of the days	99	16.8
	Nearly every day	56	9.5
**Q3: Trouble falling or staying asleep, or sleeping too much**	Not at all	220	37.2
	Several days	183	31.0
	More than half of the days	79	13.4
	Nearly every day	109	18.4
**Q4: Feeling tired or having little energy**	Not at all	76	12.9
	Several days	207	35.0
	More than half of the days	166	28.1
	Nearly every day	142	24.0
**Q5: Poor appetite or overeating**	Not at all	236	39.9
	Several days	173	29.3
	More than half of the days	102	17.3
	Nearly every day	80	13.5
**Q6: Feeling about yourself – or what you are a failure or have let yourself or your family down**	Not at all	235	39.8
	Several days	184	31.1
	More than half of the days	86	14.6
	Nearly every day	86	14.6
**Q7: Trouble concentrating on things, such as reading the newspaper or watching television**	Not at all	224	37.9
	Several days	189	32.0
	More than half of the days	101	17.1
	Nearly every day	77	13.0
**Q8: Moving or speaking so slowly that other people could have noticed**	Not at all	445	75.3
	Several days	104	17.6
	More than half of the days	25	4.2
	Nearly every day	17	2.9
**Q9: Thoughts that you would be better off dead or of hurting yourself in some way**	Not at all	434	73.4
	Several days	108	18.3
	More than half of the days	28	4.7
	Nearly every day	21	3.6

References

Schober, P.; Schwarte, L.A. Correlation coefficients: Appropriate use and interpretation. *Anesth. Analg.* **2018**, *126*, 1763–1768, doi:10.1213/ANE.00000000000028
